# Sarcoma as Second Cancer in a Childhood Cancer Survivor: Case Report, Large Population Analysis and Literature Review

**DOI:** 10.3390/medicina56050224

**Published:** 2020-05-07

**Authors:** Thinh H. Nguyen, Monish Ram Makena, Siddhartha Yavvari, Maninder Kaur, Teresia Pham, Eduardo Urias, Narendra Panapitiya, Mohamad M. Al-Rahawan

**Affiliations:** 1 Department of Pediatrics, Texas Tech University Health Sciences Center School of Medicine, Lubbock, TX 79430, USA; thinhh.nguyen@ttuhsc.edu (T.H.N.); chalia.maninder@gmail.com (M.K.); teresia.pham@ttuhsc.edu (T.P.); Eduardo.Urias@ttuhsc.edu (E.U.); Narendra.Panapitiya.MD@umchealthsystem.com (N.P.); 2 Department of Physiology, Johns Hopkins School of Medicine, Baltimore, MD 21205, USA; mmakena1@jhmi.edu; 3 Department of Epidemiology, Johns Hopkins Bloomberg School of Public Health, Baltimore, MD 21205, Usual; syavvar1@jhu.edu

**Keywords:** sarcoma, second cancer, hepatoblastoma, pediatric, SEER

## Abstract

The majority of pediatric patients are cured of their primary cancer with current advanced developments in pediatric cancer therapy. However, survivors often experience long-term complications from therapies for primary cancer. The delayed mortality rate has been decreasing with the effort to reduce the therapeutic exposure of patients with pediatric cancers. Our study investigates the incidence of sarcoma as second cancer in pediatric cancer survivors. We present a 9-year-old male who survived embryonal hepatoblastoma diagnosed at 22 months of age. At 4.5 years of age, he presented with a non-metastatic primitive neuroectodermal tumor (PNET) of the left submandibular area. He has no evidence of recurrence of either cancer for 51 months after finishing all chemotherapy and radiotherapy. We used the Surveillance, Epidemiology, and End Results (SEER) database to identify the current rate of second sarcomas in pediatric cancer survivors. Our literature review and large population analysis emphasize the impact of sarcoma as a second malignancy and provide help to physicians caring for pediatric cancer survivors.

## 1. Introduction

The current survival rate of patients with pediatric cancer is about 80%, and many of these patients are cured of their primary cancers [1]. This raised the concerns of long-term complications as a consequence of treatment for primary cancer. A recent study has shown that delayed mortality in pediatric cancer survivors drops with reducing therapeutic exposure [2].

Sarcoma as a second malignant neoplasm (SMN) in pediatrics has important clinical significance and has been extensively reported globally [3,4,5,6,7,8,9,10,11]. Findings from the Childhood Cancer Survivor Study reported a nine-fold higher risk of sarcoma among childhood cancer survivors than the general population [10]. Sarcoma as an SMN is frequently seen in survivors of specific pediatric cancers. These include leukemia, bone tumors, renal tumors, and Non-Hodgkin lymphoma [10,12]. This is likely due to the use of ionizing radiation [10,13,14,15,16,17,18,19] and DNA-damaging chemotherapeutic agents in these tumors [10,12,20,21,22,23].

We present a boy who was diagnosed with sarcoma as an SMN after surviving embryonal hepatoblastoma. The Surveillance, Epidemiology, and End Results (SEER) database was probed for the overall incidence of SMN in pediatric cancer survivors and the incidence of sarcoma as the SMN specifically. Further, we added literature review showing the global trend of sarcoma as a common SMN in childhood survivors.

## 2. Case Report

**Figure 1:**
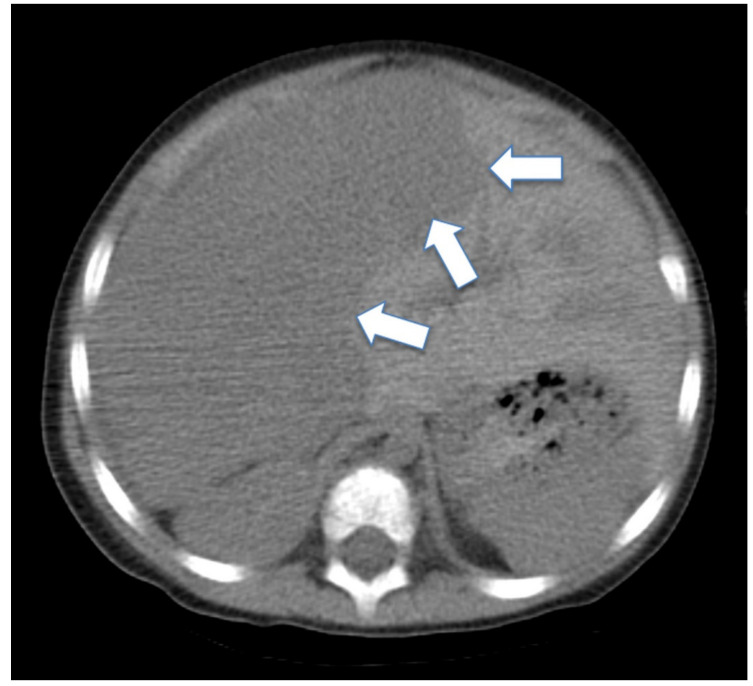
CT scan of the abdomen without contrast at the diagnosis of hepatoblastoma. It demonstrates the right liver mass measuring 10 × 10 × 15 cm. The white arrows show the line between normal and neoplastic liver tissue. The arrows are within the normal liver tissue.

**Figure 2:**
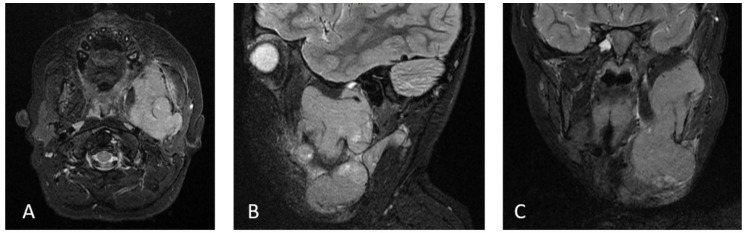
MRI of the face and mandible with and without contrast at the diagnosis of secondary PNET. The images show Short-TI Inversion Recovery (STIR). They demonstrate the mandibular mass. (**A**) Axial, (**B**) Sagittal, (**C**) Coronal.

Our patient is a 9-year-old male who presented at 22 months of age with a right-sided abdominal mass and 2-month history of lethargy, sweating, and decreased appetite. No fever or weight loss were observed. Abdominal CT and MRI revealed a very large mass of 10 × 10 × 15 cm involving most of the right lobe of the liver (Figure 1). The initial diagnosis from the frozen section favored neuroblastoma, and he received one cycle of topotecan and cyclophosphamide. The final diagnosis confirmed embryonal Hepatoblastoma, and further staging showed the involvement of segments V-VIII of the liver, putting the tumor at a PRETEXT-III. The initial AFP was over 10,000 international units per ml. There was no metastatic disease. The patient received six cycles of chemotherapy as per the Children Oncology Group (COG) protocol AHEP0731. Subsequent imaging indicated a focus of tumor remaining in the right lobe between the VII and VIII segments. The patient tolerated right hepatic lobectomy, and chemotherapy was resumed within a week after the procedure. The following cycle was complicated by a *Candida albicans* sepsis that responded to caspofungin. After completing a total of seven cycles of chemotherapy, his cumulative chemotherapy doses were as follows: Cyclophosphamide 2074 mg/m^2^, topotecan 6.2 mg/m^2^, cisplatin 600 mg/m^2^, 5-Fluorouracil 3600 mg/m^2^, vincristine 27 mg/m^2^, doxorubicin 360 mg/m^2^ (protective cardiac agent used). He had a close follow up with no evidence of relapse or organ dysfunction. Two-and-a-half years post-treatment, the patient presented with an enlarging left submandibular mass (4.7×4.2×7.2cm) (Figure 2). The mass had been present for over a month and was refractory to oral and intravenous antibiotics. The infectious disease workup, including Epstein-Barr virus (EBV), cytomegalovirus (CMV), and *Bartonella* was negative. The alpha-fetoprotein was normal at 1.9 IU/ml (normal limit 0 to 10 IU/ml). A CT scan of the mass was concerned for lymphoma with mandibular infiltration (Figure 2). A biopsy of the mass revealed PNET and the staging workup confirmed localized disease. We identified that our PNET tumor had an amplification of EWSR1-FLI1 fusion type 2 and CCND1, p16INK4a I49T, and JAK2 G571S mutations. EWSR1-FL1 fusion is found in 85 to 95% of Ewing’s sarcomas [24] and is responsible for enhancing genes involved in cell proliferation and survival [25,26]. CCND1 (cyclin D1) has been shown to be overexpressed in PNET and Ewing’s sarcoma, but not in rhabdomyosarcoma [27]. Loss of p16INK4A (inhibitor of cyclin-dependent kinases) function can lead to loss of cell cycle arrest [28]. JAK2 mutation has been detected in multiple triple-negative myeloproliferative neoplasms [29], but the role of this specific mutation in the prognosis of our patient is unknown.

He was started on induction chemotherapy per COG protocol AEWS1031. Pegylated granulocyte colony stimulating factor (Neulasta) was used and dexrazoxane was added for its cardioprotective properties, given former exposure to anthracyclines. The patient tolerated chemotherapy well with a significant reduction in the size of the mass, especially after the first cycle. He only experienced one episode of febrile neutropenia during induction. Following 12 weeks of induction, he underwent local surgical control with left hemi mandibulectomy and mandibular reconstruction. He proceeded to consolidation on the same protocol. This was composed of 12 more cycles of (VDC/IE), lasting 22 weeks. During this phase, he was treated for three episodes of bacteremia. In total, he received 25.65 mg/m^2^ of vincristine, 258.75 mg/m^2^ of doxorubicin, 10,935 mg/m^2^ of cyclophosphamide, 67,902.5 mg/m^2^ of ifosfamide, and 3871.25 mg/m^2^ of etoposide. His off-therapy evaluation revealed no residual or recurrent tumor, and his echocardiogram then showed a 65% ejection fraction and 31% fractional shortening. He is now 51 months from finishing the last chemotherapy with no evidence of recurrence. Mild asymptomatic heart failure was noted on surveillance studies at 15 months off therapy. The echocardiogram showed an ejection fraction of 50% and 24% fraction shortening. This is likely due to a high cumulative doxorubicin dose. He was referred to cardiology for the asymptomatic heart failure and to genetics for testing for cancer predisposition; however, neither has been completed by the family. He is asymptomatic and is not currently on any medications.

## 3. Literature Review

Sarcoma as an SMN in pediatric cancer survivors has been reported globally. Zakaria D et al. used Canadian Cancer Registry data, an administrative database that collects information on cancer incidence in Canada, to determine the risk of second cancer in childhood or adolescence (prior to the age of 20 years). They reported that childhood or adolescence survivors are 6.5 times more at risk for developing SMNs, and females are more prone to developing SMNs. One of the greatest risks of second cancers included soft tissue and other extraosseous sarcomas [30]. In Argentina, about 1% (34/3321) of children treated for acute leukemia and/or lymphoma developed SMNs after follow-up for 29 years. The most common SMNs reported are acute leukemia, CNS tumors, endocrinal tumors, lymphomas, and sarcomas [5]. Fidler et al. investigated the risks of subsequent primary bone cancers after childhood and adolescent cancer in 12 European countries. They reported 69,460 long term (five-year) survivors of cancer diagnosed before age 20 years, and the survivors were 22 times more likely to be diagnosed with a subsequent primary bone cancer than expected in the general population. The bone cancers were most common after retinoblastoma and sarcoma [6]. A retrospective case-series study of 10,069 pediatric cases newly diagnosed with cancer between 1980 and 2009 in Japan showed that 128 patients (1.3%) developed secondary cancers within a median follow-up of 8.4 years. Of the 128 secondary cancers, 21 (16%) cases were pathologically diagnosed as sarcomas [31]. A multicenter retrospective analysis in Korea revealed that among the 102 patients who developed SMNs, the most common SMNs included myeloid neoplasms, thyroid carcinomas, and CNS tumors [3]. MacCarthy et al. probed 1927 retinoblastoma cases from 1951 to 2004 through the United Kingdom National Registry of Childhood Tumours (NRTC), and reported that 169 SMNs. Predominant SMN cases are from a heritable form of retinoblastoma; the most common SMN was reported to be leiomyosarcoma, followed by osteosarcoma and skin melanoma [7]. The Childhood Cancer Survivor Study (CCSS) is one of the largest and most comprehensive cohort study of childhood cancer survivors, which includes 14,372 childhood cancer survivors at 26 participating clinical centers in the United States and Canada. Henderson et al. identified 108 patients with sarcomas that were diagnosed with a median of 11 years after the diagnosis of childhood cancer. The risk of sarcoma was more than nine-fold higher among childhood cancer survivors than among the general population [10]. Wilson et al. analyzed 896 childhood cancer survivors treated at the Sydney Children’s Hospital between 1972 and 1999. They reported that bone sarcoma, thyroid cancers, melanoma, and CNS malignancies were the most frequent SMNs [32]. Other reports also suggest sarcomas as common SMNs in childhood cancer survivors [33,34,35,36,37]. These studies show the global trend of sarcoma as common SMNs in childhood survivors.

Radiation, hematologic stem cell transplant, and chemotherapy were reported to cause SMNs [1]. Teepan et al. reported a dose-dependent doxorubicin-related increased risk of all solid tumors and breast cancer SMNs in the Netherland, with data obtained from a Dutch Childhood Cancer Oncology Group. The alkylating agent, cyclophosphamide, was also found to increase the sarcoma risk in a dose-dependent manner [23]. Similar results were also reported in the St. Jude Lifetime Cohort Study [38]. The retrospective cohort study across five large international sarcoma centers, Asia-Pacific (National Cancer Center, Singapore), Europe (Royal Marsden Hospital, London; Royal Orthopaedic Hospital, Birmingham; Centre Léon Bérard, Lyon), and North America (Mount Sinai Hospital, Toronto) between 2000 and 2014 revealed chemotherapy with radiotherapy influences the time-to-development of radiation-induced sarcomas [22].

## 4. SEER Database Analysis

The Surveillance, Epidemiology, and End Results (SEER) database was used for our analysis. Pediatric patients (age ≤ 16) between the years 1973–2014 were selected for primary cancer screening. We decided the cut off for the age of the first diagnosis at 16 or younger to select primary cancer in pediatric population, but there is no cap for the age at second cancer. We used the Sequence Number-Central variable to screen for patients with two different reported malignancies, which helped us to identify two separate malignant diagnoses from the same patient. We also used the ICCC3WHO variable in SEER to exclude cases that could be from a primary cancer relapse rather than second cancer developed after the treatment of primary cancer. We also used the ICCC3WHO variable to exclude patients who developed a second sarcoma after having sarcoma as their primary cancer [39]. 

## 5. Large Population Analysis from the SEER Database

Our SEER database analysis found that out of 75,665 cases of primary pediatric malignancies, 1218 (1.61%) developed SMN. The most common SMNs were leukemia, sarcoma, and thyroid carcinomas. We found that 175 out of 1218 SMNs (14.4%) were sarcomas, making sarcoma the second most common SMNs in pediatric survivors (Table 1). We also attempted to break down sarcoma into subgroups, in which osteosarcoma, fibrosarcoma, and rhabdomyosarcoma turned out to be the most common second sarcomas developing in pediatric cancer survivors (Table 2). We looked at the distribution of primary cancer in pediatric cancer survivors who developed sarcoma as an SMN. We found that retinoblastoma, lymphoid leukemia, Hodgkin lymphomas, astrocytoma, and renal tumors were the top five most common types of primary cancers. Interestingly, the time to develop second sarcomas varied from 4.1 to 15.5 years (Table 3).

## 6. Discussion

PNETs are rarely observed as SMNs [12,40,41]. To our knowledge, this is the first report of a PNET that developed in pediatric patients treated for hepatoblastoma. The role of chemotherapy and radiotherapy to treat primary malignancies has been reported to cause SMNs. This is probably further increased in survivors of SMN, who will also likely experience symptomatic organ dysfunction that may affect their quality of life and overall survival. The etiology of the subsequent PNET in our patient can be attributed to his exposure to alkylating agents; however, most therapy-related solid tumors affect cancer survivors after 5–9 years of therapy [42]. Our patient developed a PNET only 2.5 years after finishing therapy. Accordingly, we considered additional oncogenic factors. Despite multiple attempts to complete it, testing to evaluate him for cancer predisposition has not been completed.

We demonstrate that sarcoma is the second most common SMN in pediatric cancer survivors. This suggests the need for a better understanding of the development and prevention of a second sarcoma in childhood cancer survivors. We found a pattern of primary cancers among patients who developed a second sarcoma. This could be attributed to different therapies for patients with different types of primary cancers. Though the incidence of SMNs were reported to decrease in childhood cancer survivors with the time elapsed since primary malignancy diagnosis [43], we observed that pediatric survivors with astrocytomas developed a second sarcoma only after 4.1 years, while patients with Hodgkin lymphomas developed second sarcomas 15.5 years after primary diagnosis. Similar observations were highlighted by Ng A.K. et al. [44]. This supports that primary cancer therapy can influence the rate and timeline of second sarcoma development. Overall, our analysis provides a timeline that physicians can use in managing child cancer survivors. 

The limitation of our study is the missing link between different therapies and the rate of second sarcoma development. This is due to missing therapeutic and outcome variables in the SEER database. However, we attempted to establish the link of different primary cancers with the rate and timeline of the second sarcoma development. Due to the nature of our analysis, we also used the ICCC3WHO variable to exclude patients who developed a second sarcoma after having sarcoma as a primary cancer. This might limit our ability to pick up patients who truly develop second sarcoma rather than relapsed sarcoma. Still, the number of patients in this category is expected to be very small and would have minimal effect on our results. 

## 7. Conclusions

Second malignancy is a rare, but a devastating complication of cancer treatment. Pediatric cancer outcomes have seen a significant improvement over the last two decades. Cases like this raise serious considerations for clinicians and scientists with regard to the risk of SMN in pediatric cancer survivors. Our study is the first to report a PNET that developed in a pediatric survivor of hepatoblastoma. By analyzing the SEER database, we corroborated previous findings, suggesting that about 1.6% of pediatric cancer survivors develop SMNs, and 14% of those are sarcomas, similar to what the literature reports. The exact etiology of cancer, be it primary predisposition or secondary to exposures, is uncertain. The radiation and chemotherapies used in patients with pediatric cancers are carcinogenic. Our study highlights the impact of SMN in general and sarcoma specifically on the outcome of pediatric cancer survivors. It also emphasizes the need to develop novel non-DNA-damaging or alternate antineoplastic therapies to avoid the long-term potential for the development of an SMN.

## Figures and Tables

**Table 1:** Second malignant neoplasm (SMN) distribution in pediatric cancer survivors (0–16 years), analyzed using the Surveillance, Epidemiology, and End Results (SEER) database.

Cancer	Number of Cases	% of Total SMNs
Leukemia	183	15.0
Sarcoma	175	14.4
Thyroid carcinoma	166	13.6
Lymphoma	51	4.2
Astrocytoma	48	3.9
Melanoma	48	3.9
Renal carcinoma	38	3.1
Intracranial and intraspinal embryonal tumors	19	1.6
Gliomas	18	1.5
Others	472	38.8
Total	1218	100.0

**Table 2:** Distribution of the most common second sarcomas developing in pediatric cancer survivors (0–16 years), analyzed using SEER database.

Cancer	Number of Cases	% of Total Sarcoma
Other specified soft tissue sarcomas	50	28.6
Osteosarcoma	41	23.4
Unspecified soft tissue sarcomas	21	12.0
Fibrosarcoma	20	11.4
Rhabdomyosarcoma	18	10.3
Ewing sarcoma	10	5.7
Chondrosarcoma	9	5.1
Other specified malignant bone tumors	4	2.3
Unspecified malignant bone tumors	2	1.1
Total	175	100.0

**Table 3:** Primary cancer distribution for second sarcoma along with the timeline for second sarcoma development for each of these primary cancers. Time in years.

Primary Cancer	Distribution %	Age at Primary Cancer (Mean, SD, Range)	Age at SMN (Sarcoma) (Mean, SD, Range)	Years to Sarcoma from Primary Cancer (Mean, SD, Range)
Retinoblastoma	16.6	0.7, 1.1, (0–5)	14.9, 9.8, (2–41)	14.3, 9.4, (2–38)
Lymphoid leukemias	13.7	6.8, 4.9, (0–16)	14.6, 5.1, (5–22)	7.8, 4.8, (1–17)
Hodgkin lymphomas	12.0	13, 3.5, (5–16)	28.5, 10.8, (13–50)	15.5, 10.3, (3–34)
Astrocytoma	10.3	9.6, 5.5, (1–16)	13.6, 7.0, (1–26)	4.1, 5.3, (0–20)
Renal tumors	7.4	5.3, 4, (1–15)	13.5, 8.1, (4–36)	8.2, 9.2, (0–34)
